# Efficacy and safety of nintedanib in patients with non-small cell lung cancer, and novel insights in radiation-induced lung toxicity

**DOI:** 10.3389/fonc.2023.1086214

**Published:** 2023-08-10

**Authors:** Shu Yan, Shuyu Xue, Tiantian Wang, Ruihang Gao, Hanqiao Zeng, Qianmeng Wang, Xiaojing Jia

**Affiliations:** Department of Tumor Radiotherapy, The Second Hospital of Jilin University, Changchun, China

**Keywords:** nintedanib, non-small cell lung cancer, radiation therapy, radiation-induced lung toxicity, targeted therapy

## Abstract

Nintedanib is a tyrosine kinase inhibitor of fibroblast growth factor-, vascular endothelial growth factor-, and platelet-derived growth factor receptors. These three receptors promote new blood vessel formation and maintenance, which is essential for tumor growth and spread. Several trials have shown that nintedanib plays a substantial role in treating patients with non-small cell lung cancer (NSCLC) and idiopathic pulmonary fibrosis. Recently, several clinical trials of nintedanib to treat NSCLC have been reported. In this review, we focus on our current understanding of nintedanib treatment for advanced NSCLC patients and summarize the literature on using nintedanib in radiation-induced lung toxicity and the efficacy and tolerability of nintedanib.

## 1 Introduction

Lung cancer is the leading cause of cancer-related death, with a relative 5-year survival of 26.5% (1). Non-small cell lung cancer (NSCLC) accounts for over 80% of all lung cancers in patients, including squamous and non-squamous cell carcinoma. Among them, non-squamous cell carcinoma includes adenocarcinoma, large-cell carcinoma (1). Surgery, radiotherapy, and systemic therapy are the most common treatment methods for patients with NSCLC. Platinum-based combination therapy is usually recommended as initial therapy for advanced NSCLC that does not involve targeted mutations, regardless of programmed death ligand 1 (PD-L1) status (1, 2). Most patients are already in an advanced stage when first diagnosed (3). Statistical analysis showed that 25% of patients would have disease progression after first-line treatment (1). Thus, the choice of follow-up treatment drugs during disease progression is essential. Currently, docetaxel is often used as an alternative second-line treatment for patients with NSCLC (4). Nintedanib is recommended in the first set of drugs to treat idiopathic pulmonary fibrosis (IPF) owing to its unique antifibrosis effect, and its application may be similar to other interstitial lung diseases based on fibrosis (5). Recently, with the support of clinical trial data, the European Union and other countries have approved the combined use of nintedanib and docetaxel as an alternative follow-up therapeutic method for patients with advanced NSCLC who have relapsed or metastasized after first-line treatment (6).

Nintedanib is a tyrosine kinase inhibitor of fibroblast growth factor receptor (FGFR), vascular endothelial growth factor receptor (VEGFR), and platelet-derived growth factor receptor (PDGFR) (7). These three receptors promote new blood vessel formation and maintenance (8), which is necessary for tumor growth and spread. Blocking the VEGFR, PDGFR, and FGFR signaling pathways is an effective tumor therapy to inhibit tumor growth (9) To date, several trials of nintedanib in NSCLC treatment have been reported (**Table 1**), such as the LUME-lung 1 (10) and LUME-lung 2 trials (11), and the VARGDO study (14). The LUME-1 and LUME-2 trials have been completed; they demonstrate the safety and efficacy of nintedanib in NSCLC. However, the VARGDO study is ongoing, which will be explained in detail in this article.

**Table 1:** Summary of LUME-Lung 1 and LUME-Lung 2 trials.

Trial	Group	Number of patients	PFS, months (median)	OS, months (median)	Adverse Events (graded ≥ 3)		
					Diarrhea, %	Elevated alanine aminotransferase, %	Elevated aspartate aminotransferase, %
**LUME-Lung 1** (2014) (10)	Nintedanib plus docetaxel	1314 (655/659)	3.4	10.9	6.6	7.8	3.4
	Placebo plus docetaxel		2.7	7.9	2.6	0.9	0.5
**LUME-Lung 2** (2016) (11)	Nintedanib plus pemetrexed	713 (353/360)	4.4	12.0	3.5	23.3	12.1
	Placebo plus pemetrexed		3.6	12.7	1.1	7.3	1.7
**NCT02300298 (2018)** (12)	Nintedanib plus docetaxel	10	N/A	N/A	0.0	30.0	30.0
**REFRACT GFPC (2021)** (13)	Nintedanib plus docetaxel	59	2.7	6.9	3.0	0.0	0.0

OS, overall survival; PFS, progression-free survival; N/A, not applicable.

As a necessary treatment for NSCLC, thoracic radiotherapy has played a considerable role in NSCLC treatment. The incidence of radiation-induced lung toxicity (RILT) in patients with lung cancer receiving definitive radiotherapy is 5–20%. RILT occurrence severely affects the follow-up treatment and quality of life of patients. Serious RILT can even threaten the lives of patients (15). RILT is closely related to the acute immune response caused by inflammatory mediators, involving a series of cytokines, inflammatory mediators, and repair and migration of various cells (16). Many studies have considered RILT to be a complex and dynamic reaction process involving the cooperation of several factors and cells. In clinical treatments, RILT can manifest as radiation pneumonitis (RP) and radiation fibrosis (RF); these two are not separate processes but a continuous progression (17). RP to RF transition has been proven, and this transition can lead to a fatal outcome. Therefore, RILT treatment is an urgent problem for clinicians. To date, systemic corticosteroids have been commonly used to treat RILT (18). While glucocorticoids can treat RP, they cannot prevent it from developing into RF (19, 20). Therefore, finding an approach to reduce RILT symptoms is crucial. Recently, a series of validation studies on the safety and efficacy of nintedanib in RILT has been reported. Therefore, we focused on our current understanding of nintedanib treatment for advanced NSCLC patients, and by reviewing several studies that investigated nintedanib in combination with several drugs and chemotherapeutic agents, the most effective strategy with the fewest and less severe side effects could be determined to treat NSCLC. To our knowledge, there is no other review of nintedanib for RILT. We further summarized the literature on nintedanib for RILT, as well as the efficacy and tolerability of nintedanib, to provide empirical guidance for its use in future clinical work.

## 2 Nintedanib in NSCLC

In the past decades, several clinical trials demonstrated that nintedanib showed good anti-tumor activity and safe tolerance in NSCLC and other solid tumors (21, 22). In addition, some phase III clinical trials, retrospective studies, and reviews have discussed the safety and rationality of nintedanib during treatment.

### 2.1 Clinical trials

For patients with advanced NSCLC as well as with recurrence or progression in the previous treatment, especially those with adenocarcinoma as the pathological type, nintedanib combined with docetaxel has been proven to achieve better results as a follow-up treatment (10). Many patients with NSCLC are resistant to first-line platinum-based chemotherapy benefit from this treatment. Concerning second-line therapy in NSCLC, a phase III, double-blind, randomized, controlled trial, LUME-Lung 1 cohort study evaluating the safety and efficacy of docetaxel in combination with nintedanib was published (10). The LUME-Lung 1 study included 1314 patients with stage IIIB/IV NSCLC who underwent first-line chemotherapy after NSCLC diagnosis and progressed between December 23, 2008, and February 9, 2011. Patients were randomly assigned to receive docetaxel in both groups, with the difference that 655 patients received the combination of oral nintedanib and 659 patients received placebo. The treatment cycle was 21 days. On day 1 of each cycle, 75 mg/m^2^ docetaxel was administered to the patients. In the following 2–21 days, patients received a 200-mg nintedanib oral dose or placebo twice a day. Treatment was given in 3-week cycles until the patient developed intolerable side effects or disease progression, in which case the treatment was discontinued. The primary endpoint of the trial was progression-free survival (PFS), and overall survival (OS) was the secondary endpoint. After a follow-up period, the study demonstrated that patients in the docetaxel plus nintedanib group had a significantly (p=0.0019) better PFS than did patients in the placebo group (median 3.4 months vs. median 2.7 months). Moreover, NSCLC adenocarcinoma patients who progressed after receiving first-line treatment and whose progression occurred within 9 months of current treatment had significantly better OS rates after using nintedanib combined with docetaxel (206 patients, median 10.9 months) compared to those in the placebo group (199 patients, median 7.9 months). Therefore, the LUME-Lung 1 study confirmed that nintedanib combined with docetaxel could achieve better results in patients with relapsed and advanced NSCLC after first-line chemotherapy. Notably, nintedanib may have a better anti-tumor effect in non-smoking patients than in smokers (10).

The LUME-Lung 2 trial, conducted in parallel with LUME-Lung 1, was a randomized, double-blind, phase III trial reported in 2016 (11). The trial investigated whether advanced or recurrent NSCLC patients receiving nintedanib plus pemetrexed had better anti-tumor effects than did those who received pemetrexed plus a placebo. The trial included 713 patients with relapsed or refractory advanced NSCLC between December 23, 2008, and July 4, 2011. Patients were separated into two groups: nintedanib plus pemetrexed as the experimental group (n = 353) and placebo plus pemetrexed as the control group (n = 360). On day 1 of each cycle, patients in both groups received 500 mg/m^2^ pemetrexed once every 3 weeks for each cycle. On days 2–21, patients in the experimental and control groups received 200 mg nintedanib orally or placebo twice a day. PFS was considered the primary endpoint, and OS was the secondary endpoint. The pemetrexed plus nintedanib group (median 5.3 months) had significantly better PFS than did the pemetrexed plus placebo group (median 4.3 months). There was no significant difference in OS between the pemetrexed plus nintedanib group (median 12.0 months) and the pemetrexed plus placebo group (median 12.7 months) (11). The results of adverse events were similar to the outcome of LUME-Lung 1, with a higher incidence of elevated liver enzymes and diarrhea in the nintedanib group compared with placebo, as described in detail below.

However, a limitation of the LUME-Lung 2 trial is that the majority of patients included in the trial had adenocarcinoma, making it unknown how nintedanib affects other histological subtypes. In contrast to LUME-Lung 1 trial, the LUME-Lung 2 trial did not observe a significant difference in OS between the two patient groups, this is consistent with some previous studies reporting that anti-angiogenic agents combined with chemotherapy (pemetrexed or docetaxel) or erlotinib showed improvement in PFS, but not a significant improvement in OS in second-line treatment for NSCLC (23–26). Some perspectives also suggest that besides the premature termination of patient recruitment in LUME-Lung 2 trial, it may indicate that docetaxel is more suitable than pemetrexed for combination with anti-angiogenic agents.

Additionally, similar results have been reported in previous research. The feasibility of nintedanib combined with docetaxel in Japanese patients with NSCLC was investigated (12). The trial included 10 patients with advanced lung adenocarcinoma who had progressed or metastasized after first-line chemotherapy. At the end of the trial, four patients (40%) achieved a partial response, and tumors in all patients were controlled to some extent. However, considering the small sample size, this study only used descriptive analysis, and the experimental results have certain limitations. Nevertheless, these results are similar to previous experimental results, suggesting that the combination of Nivolumab and Docetaxel may benefit NSCLC patients who have progressed after platinum-based chemotherapy.

The efficacy of nintedanib in NSCLC was tested in another trial. A phase II multicenter trial involving 59 patients from 23 centers was reported in 2021. The patients included in the trial were stage IV non-squamous NSCLC patients who experienced tumor progression after receiving platinum-based combination chemotherapy. Subsequently, they received a median of four cycles of second-line therapy (nintedanib plus docetaxel). The observed PFS rate was 39.6% after 3 months of combined treatment, the median PFS was 2.7 months, and the median OS was 1.9 months. After 12 months of combined treatment, 12- month OS was 32.1%; 18-month OS was 27.6% (13).

The ongoing VARGDO study was designed to investigate the efficacy and tolerability of Vargatef (nintedanib) plus docetaxel in the routine second-line treatment of patients with locally advanced, metastatic, or locally relapsed NSCLC (14). The study included 700 patients with NSCLC who were treated with nintedanib combined with docetaxel until the tumor progressed or the patient could not tolerate it. The primary endpoint was the percentage of patients who survived 1 year after initiation of nintedanib and docetaxel (1-year survival rate) [time range: up to 24 months].

Therefore, nintedanib combined with chemotherapy can be an alternative treatment for patients with NSCLC after initial therapy progression. A phase I/II study in 2020 determined the feasibility of nintedanib combined with neoadjuvant chemotherapy in patients with NSCLC who underwent surgery (27). The primary endpoint of the study was the major pathological response (MPR). However, due to a limited number of patients, the study did not explore the relationship between MPR and other baseline patient characteristics. The findings of this study suggest that the addition of nintedanib to neoadjuvant chemotherapy did not result in an increased MPR rate. Nevertheless, the combination of neoadjuvant drugs, including cisplatin, docetaxel, and nintedanib, demonstrated sufficient safety and feasibility for resectable NSCLC. Although the combination of neoadjuvant agents showed promising results in terms of safety and feasibility, it is important to note that the addition of nintedanib to neoadjuvant chemotherapy did not significantly enhance the pathological MPR. Therefore, the use of nintedanib alone or in combination with docetaxel and platinum agents for neoadjuvant therapy is not recommended.

### 2.2 Retrospective studies

Corral et al. (28) published a retrospective analysis of 11 patients. These patients developed tumor progression after platinum-based chemotherapy and immune checkpoint inhibitor (ICI) treatment, followed by docetaxel plus nintedanib. In the analysis report, the median PFS was 3.2 months, objective response rate (ORR) was 36%, and disease control rate (DCR) was 82%.

Furthermore, a retrospective analysis of nintedanib plus docetaxel as an optional therapy for patients with advanced NSCLC was published in 2020 (29). A total of 99 patients were included, with a median PFS of 5 months (4.3–5.7 months). Fatigue (14%) and diarrhea (13%) were the most common adverse reactions with grades ≥ 3, similar to the results of the LUME-Lung 1 study.

A 2021 retrospective study in Thailand included 56 patients (30). Advanced NSCLC patients, especially with adenocarcinoma, showed the benefit of docetaxel and nintedanib combined therapy after more than 6 months of relapse after platinum-doublet chemotherapy. Another retrospective study involving 19 patients with advanced NSCLC and EGFR mutations was interesting (31). Previous studies have not examined target mutations, but this study focused on the efficacy of patients with advanced EGFR-mutated NSCLC treated with the nintedanib and docetaxel combination. The study used PFS and OS as treatment evaluation criteria. Patients were treated with this combination until disease progression became too advanced or treatment was unacceptable to the patient. The median follow-up was 11.4 months, PFS was 6.1 months (95% confidence interval (CI) 4.9–7.3), and OS was 10.1 months (95% CI 5.9–14.3). The ORR was 44.4% (23.7–66.8%), and DCR were 72.2% (49.4–88.5%). Their data indicated that the combination of docetaxel and nintedanib can be considered to be an effective treatment for EGFR TKI-resistant EGFR-mutant NSCLC.

A retrospective analysis published in 2021 reported 93 patients with advanced or metastatic pulmonary adenocarcinoma between July 2016 and February 2020 (32). In this analysis, most patients (n = 57) received first-line chemotherapy and second-line ICIs before docetaxel combined with nintedanib as third-line therapy. The remaining patients were treated with first-line chemotherapy combined with ICI, followed by nintedanib combined with docetaxel as second-line therapy (n = 10). Third-line docetaxel plus nintedanib after first-line pembrolizumab and second-line chemotherapy (n = 6) and second-line docetaxel plus nintedanib after first-line pembrolizumab treatment (n = 1) was also administered. Another 19 patients received docetaxel in combination with nintedanib as a fourth or posterior line. Most patients received docetaxel (75 mg/m^2^) on day 1 of each cycle and oral Nidanib (200 mg twice daily) on days 2–21 of each cycle. The ORR result of 93 patients was 41.4%, and DCR was 75.9%. The highest RR (ORR) was 50.0% (DCR: 82.7%) in 57 patients treated with docetaxel plus nintedanib after first-line chemotherapy and second-line ICIs. The median OS of the patients was 8.4 months after starting treatment with docetaxel plus nintedanib. PFS was not evaluated.

### 2.3 Meta-analysis

In 2014, a meta-analysis discussing the relative efficacy of second-line agents, including nintedanib plus docetaxel, which had not been directly compared in NSCLC patients with adenocarcinoma, was conducted (**Table 2**) (33). The analysis was based on nine trials conducted between January 2000 and March 2014, including 3617 patients. The research suggests that the combination of nintedanib and docetaxel provides a reliable follow-up treatment option for NSCLC patients after first-line therapy, demonstrating superior anti-tumor efficacy compared to single-agent docetaxel or erlotinib. Moreover, sensitivity analyses performed as part of the meta-analysis reveal that nintedanib plus docetaxel improves OS and PFS compared to pemetrexed monotherapy. Additionally, it demonstrates better OS compared to gefitinib. These findings from the meta-analysis support the conclusions drawn from the LUME-Lung 1 trial (10), which demonstrated that the combination of nintedanib with docetaxel provides clinical benefits over docetaxel alone as a second-line treatment for patients with advanced NSCLC of adenocarcinoma histology. Furthermore, the results suggest that this combination therapy may offer greater clinical benefits compared to erlotinib in this specific patient population.

**Table 2:** Meta-analysis of nintedanib in NSCLC.

Reference	Trial	Patient condition	Results
Popat et al. (2015) (33)	9	Adenocarcinoma	Nintedanib combined with docetaxel has clinical benefit as a second-line treatment
Popat et al. (2017) (34)	12	Adenocarcinoma	Nintedanib, in combination with docetaxel, is effective to treat NSCLC, especially in patients who are not suitable for PD-L1 therapy or have low PD-L1 expression
Vickers et al. (2019) (35)	30	EGFR mutation-positive, regardless of histology	TKI-based regimens consistently showed the the greatest benefit over docetaxel (75 mg/m^2^)
		PD-L1 expression < 5%, EGFR mutation-negative	Ramucirumab plus docetaxel and nintedanib plus docetaxel provided the most significant benefit
		PD-L1 high expression, EGFR mutation-positive	Nivolumab showed a similar benefit to the TKI

EGFR, endothelial growth factor receptor; PD-L1, programmed death ligand 1; NSCLC, non-small cell lung cancer; TKI, tyrosine kinase inhibitors.

Another meta-analysis of nintedanib plus docetaxel was published as second-line therapy in NSCLC patients with adenocarcinoma in 2017 (**Table 2**) (34). The data from these trials were discussed and summarized based on an analysis of 12 randomized controlled trials conducted between March 2014 and November 2015, which included 5521 patients. The effects of nintedanib combined with docetaxel, ramucirumab combined with docetaxel, and nivolumab in second-line treatment of patients with NSCLC adenocarcinoma were compared, and the effectiveness of these three treatment methods was generally similar. However, there were differences among the three treatment methods in each subgroup based on PD-L1 expression level. In the high-expression group, nivolumab was more effective, while in the PD-L1 low-expression subgroup, the treatment mode involving nintedanib had a better outcome.

Furthermore, a meta-analysis was published to summarize interventions for second-line treatment of patients with NSCLC (35). This analysis included 30 studies from 17 therapeutic schedules (**Table 2**). In non-squamous tumors with less than 5% PD-L1 expression and EGFR-negative tumors, ramucirumab plus docetaxel and nintedanib plus docetaxel increased the mean survival time by 2.3 months and 2.6 months, respectively, compared with docetaxel. They found that the ramucirumab-docetaxel and the nintedanib-docetaxel combinations both provided better benefits.

In the final analysis, these meta-analyses concluded that nintedanib combined with docetaxel is an alternative second-line treatment for NSCLC patients of adenocarcinoma, particularly in the low-level PD-L1 expression group. The current review highlights the role of nintedanib in NSCLC (36). We also found some cases of patients with advanced NSCLC and IPF coexistence (37–41). Patients who received nintedanib plus docetaxel as a second-line therapeutic had good results: IPF was stabilized, and the lung cancer regressed. In addition, we identified two patients with nintedanib-induced pneumonia. A case of IPF with NSCLC was reported and it was suggested that nintedanib plus prednisolone could prevent atezolizumab-related pneumonitis (42). Furthermore, the combination of nintedanib and corticosteroids reportedly had a definite treatment effect in NSCLC patients who underwent pembrolizumab-related penalties (43).

## 3 Nintedanib in other diseases

The application of nintedanib in various solid tumors has been published recently, but there are few reports on the application of nintedanib in RILT. RILT is a constantly evolving and complex process involving many drivers, including numerous pro-fibrotic, mitogenic, immunomodulatory, and pro-inflammatory mediators. Macrophages, fibroblasts, and T cells play a vital role in developing RILT and transforming growth factor β, interleukin (IL)-4, IL-13; additionally, interferon γ plays a role in this process and are further highlighted here (44). Nintedanib is approved for IPF treatment as it interferes with the active process of fibrosis and subsequently affects the disease process of IPF (45). Several trials of nintedanib applied to RILT are also ongoing.

RILT is highly common in patients with tumors who receive therapeutic radiotherapy, especially those receiving combined chemotherapy and immunotherapy. The incidence of RP in patients receiving concurrent radiotherapy and chemotherapy can reach 15–40% (46). Severe RP affects the quality of life of patients and subsequent treatment and can lead to death. Fatal RP or Grade 5 RP is a tragic event that typically occurs in ~ 2% of patients after conventional graded radiotherapy and 1% after stereotactic body radiotherapy (47). In 2019, three cases of death related to RILT were reported (47). In clinical treatment, glucocorticoid is commonly used to treat RILT. However, as a potent anti-inflammatory and immunosuppressive drug, the long-term use of glucocorticoids in large doses will increase the incidence of adverse reactions, and inappropriate use can also cause some glucocorticoid-related diseases (48, 49). Moreover, glucocorticoid does not benefit patients with radiation-induced pulmonary fibrosis (50).

A retrospective single-center study in 197 patients was published n 2022 (51). Patients were characterized as IPF, progressive fibrosing interstitial lung diseases (PF-ILD), and familial pulmonary fibrosis (FPF) and received nintedanib as a therapeutic approach from 2014–2017. The study’s objective was to evaluate the efficacy of nintedanib in these patients. The primary study results were all-cause mortality and progression-free survival, defined as the time from initiation of treatment with nintedanib to disease progression or death. In the IPF and PF-ILD subgroups, nintedanib treatment significantly reduced the forced vital capacity (FVC) decline rate. Nintedanib treatment roughly halved the FVC reduction rate in the PF-ILD subgroup compared to pre-treatment, but this result was not observed in FPF subjects. Although no significant difference in survival was observed between the subgroups, the results showed that FPF appeared less responsive to nintedanib. As of February 1, 2022, the median survival and PFS for the entire population were 999 and 683 days, respectively. At the end of the experiment, their study confirmed that nintedanib could reduce disease progression and is safe and effective in patients with IPF and IPF-ILD.

Several recent studies have established that nintedanib, an anti-VEGFR used with chemotherapy, improved survival in NSCLC. Additionally, efficacy of nintedanib has been proven in patients with IPF. However, few studies have shown the relationship between nintedanib and RILT. Wollin et al. (45) conducted a study to evaluate the antifibrotic and anti-inflammatory activity of nintedanib and suggested that the antifibrosis and anti-inflammatory activities of nintedanib may affect the clinical process of pulmonary fibrosis. In 2017, a pre-clinical experiment was published to assess whether nintedanib can reduce radiation-induced microscopic lung fibrosis (52). The experiment involved 260 C57BL/6 adult male mice irradiated with a single dose (0, 4, 6, 8, 12, 16, and 20 Gy) in the right upper lung. One week after irradiation, mice were immediately administered nintedanib by gavage (0, 30, 60 mg/kg). Micro-computed tomography (CT) images collected monthly were analyzed for lung CT density. After 39 weeks, lung tissue from mice was processed to assess the fibrosis phenotype. Quantitative noninvasive CT imaging of fibrosis development was regarded as the study’s primary endpoint, and a semi-quantitative histological examination of fibrosis after 39 weeks of irradiation was the trial’s secondary endpoint. Nintedanib reduced radiation-induced pulmonary fibrosis, and there were no other adverse reactions. In addition, the experiment also showed that using FDG positron emission tomography imaging to evaluate pulmonary fibrosis caused by coincidence had a more considerable effect.

Due to the confirmed activity of nintedanib in IPF, it has been speculated that nintedanib could be used for RILT in patients with NSCLC receiving radiotherapy. In 2020, a phase II randomized, double-blind, placebo-controlled study was conducted on nintedanib to evaluate whether nintedanib can prevent RP in NSCLC patients receiving chemotherapy and radiotherapy simultaneously (53). Patients with unresectable stage II/III NSCLC who received chemoradiation were randomly treated with 200 mg nintedanib or placebo twice daily. The primary endpoint was the standard incidence of symptomatic adverse events with grade ≥ 2 RP after chemotherapy. Secondary endpoints were survival and pulmonary function. Results showed that among the eight patients enrolled in the study (nintedanib group: n = 5; placebo group: n = 3), none of the five patients receiving nintedanib developed symptomatic pneumonia, while 67% of patients receiving the placebo had clinically significant radiotherapy-associated pneumonia. Overall, more clinically significant symptomatic events of radiation pneumonia occurred in the placebo group than in the nintedanib group. However, due to the small sample size and early study termination, this study could not conclusively prove the role of nintedanib in alleviating radiation pneumonia.

## 4 Safety and tolerability of nintedanib

Although the application of antiangiogenic drugs in cancer therapy has made considerable progress, some limitations due to the insufficient efficacy of drugs and drug resistance still exist. Antiangiogenic drugs also cause some side effects, such as toxicity, hypertension, and bleeding, limiting the effectiveness of these drugs (54). Two trials published in 2014 and 2016 demonstrated the safety and efficacy of nintedanib plus docetaxel or pemetrexed in treating NSCLC. As previously mentioned, the combination of nintedanib with docetaxel in the LUME-Lung 1 trial significantly prolonged the PFS of NSCLC patients compared to the docetaxel plus placebo group, and showed a favorable improvement in overall survival OS for adenocarcinoma patients. The 1-year OS rate in the docetaxel plus nintedanib group was 52.7% (95% CI 46.8 - 57.9), while it was 44.7% (38.9 - 49.8) in the docetaxel plus placebo group. The 2-year OS rate in the docetaxel plus nintedanib group was 25.7% (95% CI 20.5 - 30.2), compared to 19.1% (14.4 - 23.2) in the docetaxel plus placebo group. Similarly, in the LUME-Lung 2 trial, an improvement in PFS was observed in patients receiving nintedanib. It should be noted that patients receiving nintedanib were more likely to experience certain adverse reactions in both trials. However, these adverse reactions were mostly reversible and manageable through treatment interventions (10, 11, 55). Patients receiving nintedanib plus docetaxel or pemetrexed showed more frequent gastrointestinal events, including diarrhea, nausea, and vomiting, and more frequent liver enzymes elevation, primarily alanine aminotransferase (ALT), and aspartate aminotransferase (AST), than in those receiving the placebo (10, 11). Most of the above adverse reactions are treatable, and patients can recover by symptomatic treatment or by reducing the drug dosage in clinical practice (56). In LUME-Lung 1 trial, among the 652 patients in the docetaxel plus nintedanib group, 35 cases (5.4%) reported adverse events leading to death unrelated to disease progression, while in the docetaxel plus placebo group of 655 patients, there were 25 cases (3.8%) of death due to adverse events. In LUME-Lung 2 trial, the nintedanib plus pemetrexed group had 34 cases (9.8%) of death attributed to adverse reactions, while the placebo plus pemetrexed group had 44 cases (12.3%). Adverse events leading to death (≥1% incidence rate; nintedanib plus pemetrexed vs placebo plus pemetrexed) included respiratory failure (1.4% vs 1.7%), dyspnea (1.2% vs 1.4%), and pneumonia (0.6% vs 1.7%).Generally, two doses of 200 mg nintedanib is considered the recommended daily dose for combination chemotherapy in patients with advanced or metastatic NSCLC (57).

In a phase I trial published in 2015, the effect of nintedanib combined with docetaxel in previously treated Japanese patients with NSCLC was analyzed (58). They concluded that nintedanib in combination with chemotherapeutic agents showed promising results as a second-line treatment for Japanese NSCLC patients. Subsequently, an open-label feasibility study was conducted in Japan involving 10 patients (12). After regular oral treatment with docetaxel and nintedanib, the most common adverse event observed was a decrease in neutrophil count (n = 9). The second most observed adverse effects were elevated AST (n = 7) and ALT (n = 8) and gastrointestinal reactions such as constipation, diarrhea, and nausea (n = 5). The most common grade ≥ 3 adverse events were reduction in neutrophil and white blood cell counts (n = 10). However, as docetaxel can also cause a decrease in white blood cells, this myelosuppression may not be attributed solely to nintedanib. Similarly, the results of this study also indicate that a twice-daily dose of 75 mg/m^2^ docetaxel plus 200 mg nintedanib is a tolerable starting dose in patients with advanced NSCLC. Similarly, the results of this study also indicated that a starting dose of 75 mg/m2 docetaxel plus 200 mg twice daily nintedanib is well tolerated in Japanese patients with BSA < 1.5 m2 and locally advanced or metastatic lung adenocarcinoma.

The LUME-Lung 3 trial published in 2018 further confirmed this aspect. LUME-Lung 3 is a phase I study of nintedanib in NSCLC patients (59). A total of 16 patients were treated with 150 mg bid Nintedanib (n = 4) or 200 mg bid (n = 12). Treatment was given in 3-week cycles until disease progression or adverse events occurred. The study examined the efficacy of nintedanib in combination with cisplatin or gemcitabine and investigated the maximum tolerated dose (MTD) of nintedanib based on first-cycle dose-limited toxicity [DLT: drug-related non-hematologic disorder (grade ≥ 3) or hematological disorder (grade 4)]. DLT was not observed in any treated patients during the first treatment cycle. Therefore, the MTD was determined as nintedanib 200 mg bid. However, in subsequent treatment cycles, one patient in the 150-mg bid group developed grade 4 thrombocytopenia twice, and one had grade 3 thrombocytopenia. In the 200-mg bid group, one patient developed grade 3 renal failure and had grade 3 hypomagnesia. The most common adverse events associated with nintedanib occurred in the gastrointestinal tract, such as nausea, vomiting, constipation, decreased appetite, and diarrhea. In patients with nintedanib administered at a dose of 150 mg bid, four patients presented with nausea (100%, grade < 3), vomiting occurred in two of them (50%, grade < 3), and constipation in one patient (25%, grade < 3). Moreover, four patients had a decreased appetite (100%, grade < 3), and four patients had diarrhea (100%), including one patient (25%) with grade 3 or above diarrhea. In patients with nintedanib administered at a dose of 200 mg bid, nausea occurred in nine patients (75%, grade < 3), vomiting in eight patients (66.7%, grade < 3), constipation in eight patients (66.7%, grade < 3), decreased appetite in five patients (41.7%, grade < 3), and diarrhea in five (41.7%, grade < 3). Hematological adverse events in this study were considered to be expected with cisplatin/gemcitabine treatment and not classified as DLT. Finally, five patients had a partial response, and eight had stable disease, with an overall response rate of 31.3% and a disease control rate of 81.3%. The median OS was 6.7 months. The 6-month OS rate was 69%. The median PFS was 4.2 months, and the 6-month PFS rate was 25%. In this trial, similar to previous trials, the recommended dose of 200 mg nintedanib twice daily orally had some safety and manageable adverse effects when combined with cisplatin or gemcitabine.

We also summarized some studies of nintedanib to treat IPF. A phase II trial evaluating the safety and efficacy of four different oral doses of nintedanib (BIBF 1120) versus placebo in patients with IPF was published in 2011 (60). The study involved 432 patients with IPF who were randomly assigned to receive nintedanib or a placebo. Both groups received four doses of nintedanib (50 mg once daily, 50 mg twice daily, 100 mg once daily, or 150 mg twice daily) or placebo. The primary endpoint was the annual rate of decline in FVC. Secondary endpoints included acute exacerbation, quality of life (measured by the St. George Respiratory Questionnaire), and total vital capacity. Over 12 months of observation, FVC decreased by 0.06 L per year in the group receiving 150 mg BIBF 1120 twice daily compared to 0.19 L per year in the placebo group. Analysis of trial data showed that a twice-daily dose of 150 mg of nintedanib had better benefits than did the placebo group.

We also reviewed two 52-week randomized, double-blind, replicated phase III trials of nintedanib in patients with IPF (INPULSI-1 and INPULSI-2) (61). This study investigated whether nintedanib is safe and advantageous in treating IPF by observing the degree of change in FVC after treatment with nintedanib or placebo in two randomly assigned patient groups with IPF. The trials included 1066 patients (INPULSI-1: n = 513; INPULSI-2: n = 548) between May 2011 and September 2012, who were randomly separated into two groups at a ratio of 3:2 (INPULSI-1 nintedanib group: placebo group = 309:204; INPULSI-2 nintedanib group: placebo group = 329:219). Patients received 150 mg nintedanib or a placebo twice daily to assess the annual rate of decline in FVC over a 52-week treatment period. The most common adverse event in the nintedanib group was gastrointestinal reactions, particularly diarrhea (INPULSI-1: n = 190, 61.5%; INPULSI-2: n = 208, 63.2%). Most patients who developed diarrhea in the nintedanib group reported events of mild or moderate intensity (93.7% in INPULSI-1 and 95.2% in INPULSI-2). Patients in the nintedanib group had higher levels of liver enzymes than those in the placebo group. In INPULSI-1, 15 patients (4.9%) in the nintedanib group and 1 patient (0.5%) in the placebo group had levels of AST, ALT, or both that were three times or more above the upper limit of the normal range. In INPULSI-2, 17 patients (5.2%) in the nintedanib group and 2 patients (0.9%) in the placebo group exhibited this increase. Concerning severe adverse effects, the proportion in the nintedanib group was similar to that in the placebo group. In addition, cardiovascular events were uncommon in both groups with five patients in the nintedanib group and one in the placeobo group with a mycocardial infarction in both trials. In conclusion, nintedanib significantly reduced the rate of FVC decline in IPF patients over 52 weeks in both trials. Mild gastrointestinal reactions were the most common adverse reaction in the nintedanib group. Similar to nintedanib for NSCLC, elevated liver enzymes are not uncommon in nintedanib treatment, and the elevated liver enzymes rate was higher with nintedanib for IPF than with placebo. As for cardiovascular events, although rare, they occurred at similar rates in the nintedanib and placebo groups. Although more patients with myocardial infarction were observed in the nintedanib group, available data do not indicate a valid association between this adverse event and nintedanib. Therefore, additional data are needed to explore this aspect. In a 2020 review study, Ameri et al. (62) discussed whether nintedanib increases the risk of cardiovascular disease. This conjecture is reasonable because nintedanib can block VEGFR, which may reduce platelet activity and leukocyte adhesion, thus increasing the hemorrhage and thrombosis risk. In addition, nintedanib interferes with platelet cytogenesis by blocking PDGF-α and PDGF-β, leading to thrombocytopenia (63). The clinical trials and research summary has raised doubts about whether nintedanib will increase the probability of myocardial infarction (62). However, this conjecture cannot be confirmed due to the lack of myocardial infarction records after using nintedanib in clinical practice.

In a 2022 study, nintedanib adverse events were also reported as primarily gastrointestinal, especially diarrhea (64). During clinical treatment, symptomatic treatment and dose adjustment should be adopted to deal with adverse events. Treatment with nintedanib immediately after IPF diagnosis is beneficial. In addition, another study also discussed adverse events when using nintedanib (65). This reporting system analyzed data from 633 patients in the INBUILD trial, evaluated whether nintedanib could be used to treat fibrotic interstitial lung disease (ILDs), and summarized adverse events that occurred during treatment. The patients with fibrointerstitial fibrosis were divided into two groups: 332 in the nintedanib group and 331 in the placebo group. By assessing adverse events and dose adjustments for adverse events over 52 weeks of nintedanib administration, 22.0% of adverse events resulted in treatment interruption in patients receiving nintedanib and 14.5% in patients receiving a placebo. The most common adverse event was diarrhea, reported by 72.3% of patients in the nintedanib group and 25.7% in the placebo group. They concluded that diarrhea was the most common adverse event, and patients were likely to have dose reduction or even treatment discontinuation due to severe diarrhea (66).

Ogura et al. (67) further analyzed data from the INBUILD trial on 108 Japanese patients. On one hand, the proportion of patients with ILD progression or death throughout the trial was 38.5% (n = 20) in the nintedanib group versus 55.4% (n = 31) in the placebo group. The proportion of patients with acute exacerbation or death was 7.7% in the nintedanib group (n = 4) versus 28.6% in the placebo group (n = 16). Compared with a placebo, nintedanib reduced the risk of disease progression and death in PF-ILD patients but not in IPF patients. On the other hand, patients treated with nintedanib were more likely to experience nausea, vomiting, loss of appetite, weight loss, abdominal pain, and liver adverse events than those treated with a placebo. The most common adverse event was diarrhea, which was more frequent in the nintedanib group (n = 44, 84.6%) than in the placebo group (n = 20, 35.7%). In most cases, severity of diarrhea was grade 1 (nintedanib, n = 32; placebo, n = 19) or grade 2 (nintedanib, n = 9; placebo, n = 1). Grade 3 events were rare (nintedanib, n = 3; placebo, n = 0), and none of the patients developed grade 4 diarrhea. A total of 11 patients (21.2%) in the nintedanib group had liver enzymes(ALT and AST) increased ≥ 3 times the upper limit of the normal range compared with no patients in the placebo group. Notably, because nintedanib is a VEGFR inhibitor, its use may increase the risk of bleeding in patients. However, actual data showed that patients receiving nintedanib did not have a higher incidence of bleeding adverse events than those receiving a placebo. Similarly, concerning adverse cardiovascular events, the rate of adverse cardiovascular events was not higher in the nintedanib group compared to that in the placebo group.

We also evaluated information regarding the safety evaluation of nintedanib in other diseases. In 2019, a phase Ib trial involving patients with advanced solid tumors examined the possible adverse effects of nintedanib in combination with bevacizumab (68). In the planned treatment of nintedanib combined with bevacizumab, there were no dose-limiting toxicities in 18 participating patients, and the most common adverse reactions were fatigue (n = 15,83%, grade 1–3) and diarrhea (n = 11,61%, grade 1–2). Nintedanib combined with bevacizumab had a good effect in treating solid tumors. Moreover, when combined with pembrolizumab, nintedanib was recommended at 150 mg twice daily (69). A multicenter, open-label, phase I/randomized phase II study was conducted, which further explored the safety profile of drugs used to treat patients with advanced hepatocellular carcinoma in Europe. Furthermore, whether nintedanib could be used as a targeted agent like sorafenib to treat advanced hepatocellular carcinoma was investigated (70). Among patients receiving nintedanib and sorafenib, the incidence of nausea was 48.4% (n = 30) and 29%(n = 9), and the incidence of vomiting was 38.7% (n = 24) and 29%(n = 9). Among patients who developed nausea or vomiting after receiving nintedanib, 1 patient (1.6%) had ≥ grade 3 nausea and 2 patients (3.2%) had ≥ grade 3 vomiting. No grade 3 or higher nausea or vomiting was observed in patients treated with sorafenib. The incidence of nausea and vomiting was higher in patients using nintedanib than in those using sorafenib. For patients treated with nintedanib and sorafenib, mean TTP (Time-to-progression) was 5.5 vs. 4.6 months, mean PFS was 5.3 vs. 3.9 months, and mean OS was 11.9 vs. 11.4 months. The two drugs showed similar efficacy against tumors.

A phase II study published in 2016 evaluated the efficacy and safety of nintedanib in patients with relapsed SCLC (71). This study showed that nintedanib was well tolerated by the patients, although it showed only limited activity in treating SCLC. However, due to the lack of clinical evidence, more trials are needed to confirm the safety and efficacy of nintedanib in SCLC.

In general, although nintedanib may have some adverse effects in treating NSCLC, the therapeutic effect of nintedanib for NSCLC is positive. When combined with chemotherapy drugs, nintedanib has demonstrated a significant improvement in PFS. The most common additional adverse reactions associated with nintedanib include gastrointestinal effects such as nausea, vomiting, constipation, decreased appetite, and diarrhea, as well as elevated liver enzyme levels. Hematological toxicities, such as decreased white blood cell and neutrophil counts, are not uncommon due to the combined use of nintedanib with chemotherapy drugs. Although nintedanib possesses activity in blocking VEGFR, cardiovascular adverse events are rare, and existing evidence does not establish a clear association between nintedanib and cardiovascular events. Moreover, most adverse events are manageable and can be effectively controlled with specific therapies or by reducing the nintedanib dose. When nintedanib is combined with other NSCLC chemotherapeutic agents, an oral dose of 200 mg twice daily is safe. When nintedanib is combined with targeted agents, a dose of 150 mg twice daily is recommended. For IPF treatment, the recommended oral nintedanib dose is 150 mg twice daily. As for RILT in NSCLC patients receiving radiotherapy, there are not enough trials to determine the nintedanib dosage, although nintedanib can be used as an anti-tumor agent and may have an effect on RILT.

## 5 Conclusion

In recent years, nintedanib has emerged as a therapeutic agent for IPF and NSCLC. Due to the wide use of radiotherapy in patients with NSCLC, RILT produced by multiple factors often appears in patients with lung cancer who have received thoracic radiotherapy. Therefore, a drug that can both treat NSCLC and prevent RILT would be valuable. Studies have demonstrated the safety and efficacy of nintedanib in advanced NSCLC, and although there are few clinical studies of nintedanib for RILT, the new research is valuable. At present, it is essential to continue the search for drugs that can treat RILT. Lastly, research should continue using cell lines, animal models, and clinical trials to explore effective and appropriate prevention and treatment of RILT.

## Author contributions

SY, SX, TW, RG, HZ, QW, and XJ contributed substantially to the conception, drafting, editing, and final approval of this manuscript.
